# Supplementary material for: Effects of horizontal displacement and inter-character spacing on transposed-character effects in same-different matching
Source: PLoS One. 2022 Mar 21;17(3):e0265442. doi: 10.1371/journal.pone.0265442 (PMC8936455; doi:10.1371/journal.pone.0265442)
Supplement: S1 File — (PDF) [file pone.0265442.s001.pdf]

Effects of horizontal displacement and inter-character spacing on transposed-character effects in same-different matching

Stéphanie Massol[1] & Jonathan Grainger[2, 3, 4]

Supporting Information: S1 File.

Experiment 1 - RT analyses

In Experiment 1, statistical analyses were performed only over the “different” trials, since there was no experimental manipulation of Type of change within the set of “same” trials. Trials associated with an incorrect response were excluded (36.46% of the data) as well as RTs shorter than 300 ms or longer than 1300 ms (1.89% of the data). RTs were transformed (-1000/RT) to reduce the skewness in the distribution. RTs were analyzed using models including items and participants as crossed random effects (including by-item and by-participant random intercepts; [39] and with random slopes [40]. The logistic mixed-effects model included Type of Character (letter, digit, symbol), Target Location (central vs. displaced) and Type of Change (transposition vs. substitution) as fixed-factors. The models were fitted with the lme function from the lme4 package [41] in the R statistical computing environment [42]. The maximal random effects structure that converged was one including by-participant and by-item random intercepts, as well as by-participant random slopes for Type of Character. The following analyses were conducted taking the letter string condition as reference for the Type of Character factor, the central condition as reference for the Target Location factor, and the substitution condition as reference for the Type of Change factor. Mean RTs for each of the experimental conditions are presented in Fig S1.

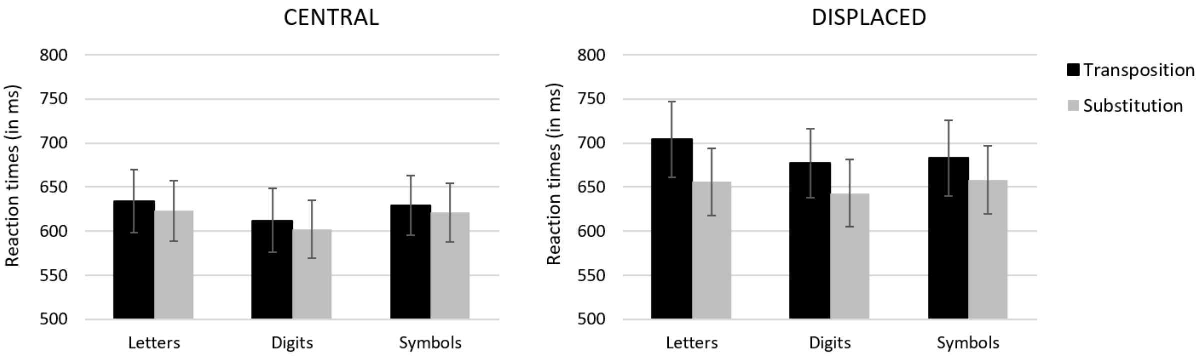

Fig S1. Reaction times (in ms) for each type of stimulus in the transposition and in the substitution conditions when the target string was presented centrally (left panel) and when it was presented displaced two character spaces to the right or left (right panel) in Experiment 1. Error bars represent within-participant 95% CIs [35]. Note: Mean RTs for the "same" trials were 574 ms, 589 ms and 581 ms for the letter, digit and symbol strings, respectively.

RTs were shorter for digit strings than for letter strings (634 ms vs. 654 ms respectively; $b = -0.04$, $SE < 0.01$, $t = -4.50$), whereas there was no significant difference between letter strings and symbol strings (654 ms vs. 648 ms respectively; $t = 0.51$). Furthermore, shorter RTs were observed when the target was presented at the center of the screen than when it was shifted by two characters to the left/right (620 ms vs. 670 ms respectively; $b = 0.08$, $SE < 0.01$, $t = 8.60$). There was also a significant effect of Type of Change, with shorter RTs in the substitution condition than in the transposition condition (634 ms vs. 657 ms respectively; $b = 0.03$, $SE < 0.01$, $t = 3.61$). Importantly, the interaction between Target Location and Type of Change was significant ($b = 0.05$, $SE = 0.01$, $t = 3.57$), reflecting the fact that the effect of Type of Change was greater with displaced targets. The Type of Character (letters vs. digits) x Target Location interaction as well as the Type of Character (letters vs. symbols) x Target Location interaction were not significant ($ts < |0.8|$). All other interactions were not significant.
