# Supplementary material for: Effects of horizontal displacement and inter-character spacing on transposed-character effects in same-different matching
Source: PLoS One. 2022 Mar 21;17(3):e0265442. doi: 10.1371/journal.pone.0265442 (PMC8936455; doi:10.1371/journal.pone.0265442)
Supplement: S2 File — (PDF) [file pone.0265442.s002.pdf]

Effects of horizontal displacement and inter-character spacing on transposed-character effects in same-different matching

Stéphanie Massol[1] & Jonathan Grainger[2, 3, 4]

Supporting Information: S2 File.

Experiment 2 - RT analyses

In the same way as Experiment 1, trials associated with an incorrect response were excluded (30.59% of the data) as well as RTs shorter than 300 ms or longer than 1300 ms (1.19% of the data). RTs were transformed (-1000/RT) to reduce the skewness in the distribution. RTs were analyzed with linear mixed-effects models using in R [42]. The models included by-item and by-participant random intercepts [39], and Type of Character, Type of Change and Space as fixed effects. The following analyses were conducted taking the letter string condition as reference for the Type of Character factor, the normal condition as reference for the Space factor, and the substitution condition as reference for the Type of Change factor. Mean RTs for each of the experimental conditions are presented in Fig S2.

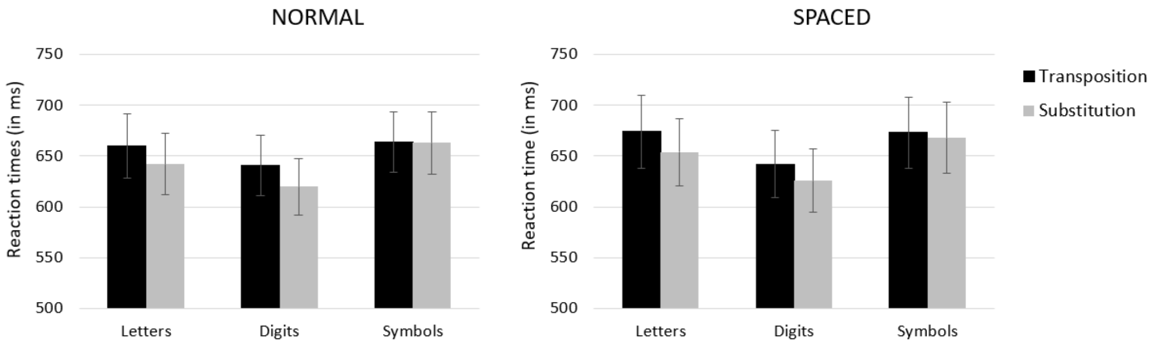

Fig S2. Reaction times (in ms) for each type of stimulus in the transposition and in the substitution conditions when the target string was presented centrally (left panel) and when it was presented displaced two character spaces to the right or left (right panel) in

Experiment 2. Error bars parentheses represent within-participant 95% Cis [35]. Note: Mean RTs for the "same" trials were 598 ms, 584 ms and 605 ms for the letter, digit and symbol strings, respectively.

RTs were shorter for digit strings than for letter strings (633 ms vs. 658 ms respectively; $b = -0.04$, $SE < 0.01$, $t = -4.70$). RTs were also shorter for letter strings than for symbol strings (658 ms vs. 668 ms respectively; $b = 0.05$, $SE = 0.01$, $t = 4.92$). Furthermore, longer RTs were observed when the target had an extra-spacing between each of its constituent characters than when the target was normally presented (657 ms vs. 649 ms respectively; $b = 0.01$, $SE < 0.01$, $t = 2.16$). There was also a significant effect of Type of Change, with shorter RTs in the substitution condition than in the transposition condition (646 ms vs. 660 ms respectively; $b = 0.04$, $SE < 0.01$, $t = 5.75$). The Type of Character (letters vs. symbols) x Type of Change interaction was significant ($b = -0.03$, $SE = 0.01$, $t = -2.76$). Subsequent pairwise comparisons were run with testInteractions function including the 3 levels of Type of Character (letters, digits, symbols) and the 2 levels of Type of Change (transposition vs. substitution). The effect of Type of Change was significant for letter and digit strings and marginal for symbol strings (letters: $\chi^2(1) = 53.71$, $p < .001$, digits: $\chi^2(1) = 58.39$, $p < .001$, symbols: $\chi^2(1) = 3.31$, $p = .068$). All other interactions were not significant.
